# Streamlining the annotation process by radiologists of volumetric medical images with few-shot learning

**DOI:** 10.1007/s11548-025-03457-3

**Published:** 2025-06-25

**Authors:** Alina Ryabtsev, Richard Lederman, Jacob Sosna, Leo Joskowicz

**Affiliations:** 1 School of Computer Science and Engineering, The Hebrew University of Jerusalem, Jerusalem, Israel; 2 Dept. of Radiology, Hadassah University Medical Center, Jerusalem, Israel

**Keywords:** Medical image detection and segmentation, Few-shot learning, Deep learning, Annotation efficiency

## Abstract

**Purpose:**

Radiologist’s manual annotations limit robust deep learning in volumetric medical imaging. While supervised methods excel with large annotated datasets, few-shot learning performs well for large structures but struggles with small ones, such as lesions. This paper describes a novel method that leverages the advantages of both few-shot learning models and fully supervised models while reducing the cost of manual annotation.

**Methods:**

Our method inputs a small dataset of labeled scans and a large dataset of unlabeled scans and outputs a validated labeled dataset used to train a supervised model (nnU-Net). The estimated correction effort is reduced by having the radiologist correct a subset of the scan labels computed by a few-shot learning model (UniverSeg). The method uses an optimized support set of scan slice patches and prioritizes the resulting labeled scans that require the least correction. This process is repeated for the remaining unannotated scans until satisfactory performance is obtained.

**Results:**

We validated our method on liver, lung, and brain lesions on CT and MRI scans (375 scans, 5933 lesions). It significantly reduces the estimated lesion detection correction effort by 34% for missed lesions, 387% for wrongly identified lesions, with 130% fewer lesion contour corrections, and 424% fewer pixels to correct in the lesion contours with respect to manual annotation from scratch.

**Conclusion:**

Our method effectively reduces the radiologist’s annotation effort of small structures to produce sufficient high-quality annotated datasets to train deep learning models. The method is generic and can be applied to a variety of lesions in various organs imaged by different modalities.

**Supplementary Information:**

The online version contains supplementary material available at 10.1007/s11548-025-03457-3.

## Introduction

Deep learning (DL) models have become the method of choice for the automatic detection and segmentation of structures in medical images. Supervised learning methods require training datasets with radiologists' annotations. The most popular DL architecture, U-Net [1], yields good results for pixel-level classification across different imaging modalities [2]. Its successor, the nnU-Net [3], automates various model construction tasks, i.e., architecture configuration, training and test time augmentations, and selection of hyperparameters. It achieved state-of-the-art results for a variety of structures and tasks [4]. However, hundreds of annotated images are typically required to train these models to achieve the desired performance [5]. Obtaining the annotations constitutes a significant bottleneck, as the annotation requires expertise and is very time-consuming, especially for volumetric medical images (scans).

Recent research has focused on developing image annotation methods to expedite the labeling task [6–9]. Some use pre-trained pixel-level classification models to identify potential regions of interest in the scan—the user then is only required to accept, reject, or modify the computed labels, which is expected to require less effort than manual annotation. Bangert et al. [6] describe an active learning method that selects the most informative images for manual annotation. After enough images are annotated, the model automatically labels the remaining images for review by a radiologist. Using 16% of the images of a dataset (8,045 of 50,286 images), the method achieved 85% accuracy for cellular breast cancer classification. However, this method is limited to image-level classification and has not been tested for detection and segmentation of structures in scans. Zhang et al. [7] introduce SAMAug, based on the segment anything model (SAM) [8]. SAM generates segmentation and boundary prior maps from user prompts, which are combined to create training datasets for supervised DL models. SAMAug achieved a Dice score of 0.79–0.85 on three types of optical image datasets with a few tens of training examples. This method is limited to 2D images and requires user inputs for each image, which is labor-intensive for 3D images and for small structures, e.g., lesions.

Few-shot learning (FSL) is a promising paradigm for addressing the labeling issue. Using prior knowledge, it utilizes a few annotated scans to generalize a new task and generate labels for a larger unlabeled dataset [9]. Meta-learning, a central approach in FSL, focuses on learning across tasks to generalize to new tasks with minimal labeled data. Several meta-learning-based FSL methods have been recently developed. Wang et al. [10] introduce the prototype alignment network (PANet), based on prototypical networks. Prototypical networks represent images as points in an embedding space in which similar objects are close to each other, while different objects are far apart. PANet separates knowledge extraction and labeling using prototypes from the support set for nonparametric pixel-wise nearest-neighbor matching. It achieved an intersection-over-union (IoU) score of 70.7 on the 5-shot segmentation task on the PASCAL dataset [11].

Ouyang et al. [12] present ALPNet (adaptive local prototype pooling network), based on the PanNet model. It uses local and global prototypes to segment large structures, i.e., organs in abdominal CT scans. It achieved a Dice score of 0.82 for liver segmentation with 5-shot learning. Butoi et al. [13] describe UniverSeg, an FSL meta-learning-based model for voxel-level classification of structures in scans. It uses a cross-block mechanism embedded into a U-Net-like architecture. The UniverSeg model, pre-trained on over 22,000 scans from various modalities and anatomical structures from 53 open-access medical images datasets, achieved a mean Dice score of 0.71 on a variety of anatomical structures. UniverSeg has become the state-of-the-art FSL model due to its generalization capability, which does not require structure-specific training. Nonetheless, UniverSeg is only applicable to 2D images and large structures, e.g., organs. None of the FSL methods was designed or tested for the detection and segmentation of small pathologies, e.g., lesions in scans.

This paper presents a novel method for streamlining the process of annotating volumetric medical image datasets used to train fully supervised deep learning voxel-level lesion classification models. The method focuses on obtaining annotations for scans with small structures and from various imaging modalities on individual slides (2D images) of scans. The method is an end-to-end pipeline that inputs a small dataset of labeled volumetric scans and a large dataset of unlabeled scans, and outputs a high-quality labeled dataset with which a fully supervised model is trained until it yields satisfactory performance. Our method reduces the estimated correction effort of radiologists by using a few-shot learning model to generate initial labels for the new structure of interest using an optimized support set of scan slice patches and by prioritizing the resulting labeled scans that require the fewest corrections.

The contributions of this paper are: (1) a new method for the generation of high-quality labeled scans and a fully supervised model that minimizes the expected manual correction effort and achieves a desired performance on an independent test set; (2) a policy for selecting the most representative labeled patches for the support set of a few-shot learning model; (3) a strategy for prioritizing a subset of scans with computed labels for review and correction by a radiologist with reduced effort; (4) metrics for estimating the effort required by a radiologist to correct the computed lesions, quantified by the number of mouse clicks required for the correction; (5) extensive experimental evaluation of the method in three use-case scenarios: detection and segmentation of liver metastases in abdominal contrast-enhanced CT scans, lung metastases in chest CT scans, and brain metastases in brain MRI scans.

## Method

**Fig. 1:**
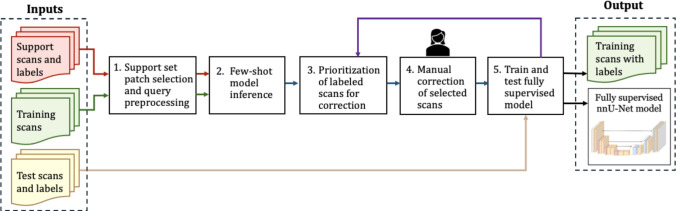
Overview of the method. The inputs are: a small support dataset of annotated scans (red), a large dataset of unannotated training scans to be used as query scans (green), and a test dataset of annotated scans (yellow). The outputs are a large, high-quality dataset of annotated scans (green) and a nnU-Net model for voxel-level lesion classification trained on those scans. The steps are: (1) support set patch selection and query scans pre-processing; (2) few-shot learning inference; (3) prioritization of the scans for correction based on the computed labels; (4) manual correction of a subset of the scan labels by a radiologist; (5) training of a nnU-Net model on the support and query labeled datasets and testing its performance on the test dataset. This process is repeated when needed for the remaining unlabeled scans until the trained nnU-Net model yields satisfactory performance on the test dataset

Our method streamlines the process of manual annotation of small pathologies, e.g., lesions, in scans. The goal is to obtain sufficient, high-quality, expert-validated labeled data to train a fully supervised deep learning model. The method uses a pre-trained few-shot learning model with a small support set of manually annotated scans to compute initial voxel-level labels for a larger set of unannotated scans. A subset of these scans is then selected for review and correction by a radiologist. By prioritizing the labeled scans for corrections, the method minimizes the correction effort while increasing the labels representativity. A fully supervised model is then trained on the scans with corrected labels and the support set scans and tested on an independent annotated dataset. The process is repeated for the remaining unannotated scans until satisfactory performance is obtained. The method is generic and can be applied to various volumetric medical imaging modalities, organs, and pathologies. Figure 1 shows the pipeline.

Our method is based on two key novel ideas. The first is a policy for selecting the most representative labeled patches for the support set. Since lesions have various shapes, patterns, textures, contrasts, and appear in various organs, it is important to select a support set that reflects the distribution of the lesions. The second is a strategy for prioritizing the subset of scans with computed labels for review and correction by a radiologist with minimal effort and maximal performance when using the corrected scans to train a fully supervised model.

### Support set patch selection and pre-processing of query scans

This step prepares the support and query scans for the few-shot learning model and builds the support set of patches based on empirical heuristics. First, the scans in both datasets are pre-processed by pre-set intensity clipping and min–max normalization to scale the values to [0,1]. Next, 128 × 128 patches with a ½ stride are extracted from each support and query set scan. Positive patches, e.g., those with a minimum number of positive lesion pixels (> 30 pixels), are selected from the generated support patches.

Next, a fixed-size subset of patches is drawn from the resulting set of patches with a k-means clustering-based strategy that ensures diversity of patterns of lesion parts. The policy proceeds as follows. First, binary flattened vectors are generated for all the positive patches. Then, k-means clustering is performed on them with *k* = 100 clusters. This number of clusters was selected since we empirically found that it enhances the generalization performance of the UniverSeg model while incurring in a negligible addition to computation cost. Next, the cluster containing the highest number of patches, typically representing irrelevant, infrequent, or background patterns, is excluded. The final patches subset is then sampled proportionally from the remaining 99 clusters. This results in a set of labeled support set patches and an unlabeled query set of patches. Inference is the performed by the FSL model on the resulting query and support sets of patches.

### Few-shot model inference

This step computes initial voxel-level lesion labels for the query set using the selected labeled support patch set. We use the weights of the pre-trained UniverSeg FSL model, which were pre-computed on open-source datasets for various tasks, structures of interest, and imaging modalities [13]. The UniverSeg FSL model is used to compute the labels for the query set patches alongside the generated support set, with a ½ stride overlap for each query scan slice. The resulting labels for each patch in each scan slice are then combined into 3D lesion segmentations corresponding to the scan by identifying the connected components in 3D. Finally, the produced labels are post-processed by filling in holes and region-based morphological operations for removing very small lesions ($\le$ 30 voxels, ~ 3 mm).

### Prioritization of computed scan labels for correction

This step selects a subset of the resulting labeled scans for review and correction by a radiologist, which may require minimal corrections.

The prioritization policy is designed to rank the scans with the computed labels according to the number of detected lesions whose diameter is $\ge$ 10 mm or larger. Scans with more lesions $\ge$ 10 mm are prioritized higher in descending number of lesions. Scans with no lesions $\ge$ 10 mm are ranked lower, with no order preference. Scans without lesions are ranked lowest. This is because scans without large lesions tend to be less informative clinically, as they often indicate stable or slow disease progression and fewer lesion distributions. In contrast, scans with larger lesions are generally segmented more accurately by FSL models, reducing the need for extensive manual corrections. For example, according to this policy a scan with four lesions $\ge$ 10 mm will be ranked higher than a scan with two lesions $\ge$ 10 mm and ten lesions < 10 mm. The number of scans *M* to correct is pre-determined, usually set to 10–30.

### Manual correction of the prioritized subset of labeled scans

The review and correction of the computed lesion labels on the selected scans by a radiologist proceeds as follows: each slice of each selected scan is reviewed by overlaying the computed lesion labels on the original slice. Wrongly identified lesions (false positives) are removed with a single click inside the computed lesion. The voxels in the lesion connected component are automatically unlabeled. Missed lesions (false negatives) are added by delineating their contour with one mouse click per contour pixel. Correctly identified lesions (true positives) whose contours are partially incorrect are revised by adding and deleting pixel contours, with one mouse click per erroneous pixel on the lesion contour and automatic addition or deletion of the interior pixels.

### Detection and segmentation of lesions with a fully supervised model

This step trains a fully supervised model to obtain an intermediate model that produces better voxel-level lesion labels than the FSL model on the remaining unlabeled scans. The model is trained with the corrected labeled scans and the labeled support set scans. The performance of the resulting model is quantified with the labeled test set scans. If the performance is acceptable, the process stops and outputs the labeled datasets and the trained model. Otherwise, the process is repeated for the remaining unannotated scans until satisfactory performance is obtained.

For inference, we use the supervised 3D nnU-Net [3]. It automatically preprocesses the training data and select the hyperparameters, the augmentations, and the U-Net configuration. Training is performed using a fivefold cross-validation with Dice and cross-entropy loss functions. Various augmentations, e.g., affine transformations, mirroring Gaussian noise, blurring, brightness, contrast, resolution reduction, and Gamma correction, are used. The learning rate is adjusted during each iteration with a polynomial function.

## Experimental setup

**Datasets:** scans of patients with liver, lung, and brain metastases were collected from two Hadassah University Medical Centers (Jerusalem, Israel). These include contrast-enhanced chest and venous-phase abdominal CT scans (Philips CT Brilliance iCT, Canon CT Aquilion Prime SP, GE CT Optima 660) and axial 3D T1W-Gadolinium enhanced brain MRI scans (Philips Ingenia, GE Voyager, Siemens Avanto Fit).

**Fig. 2:**
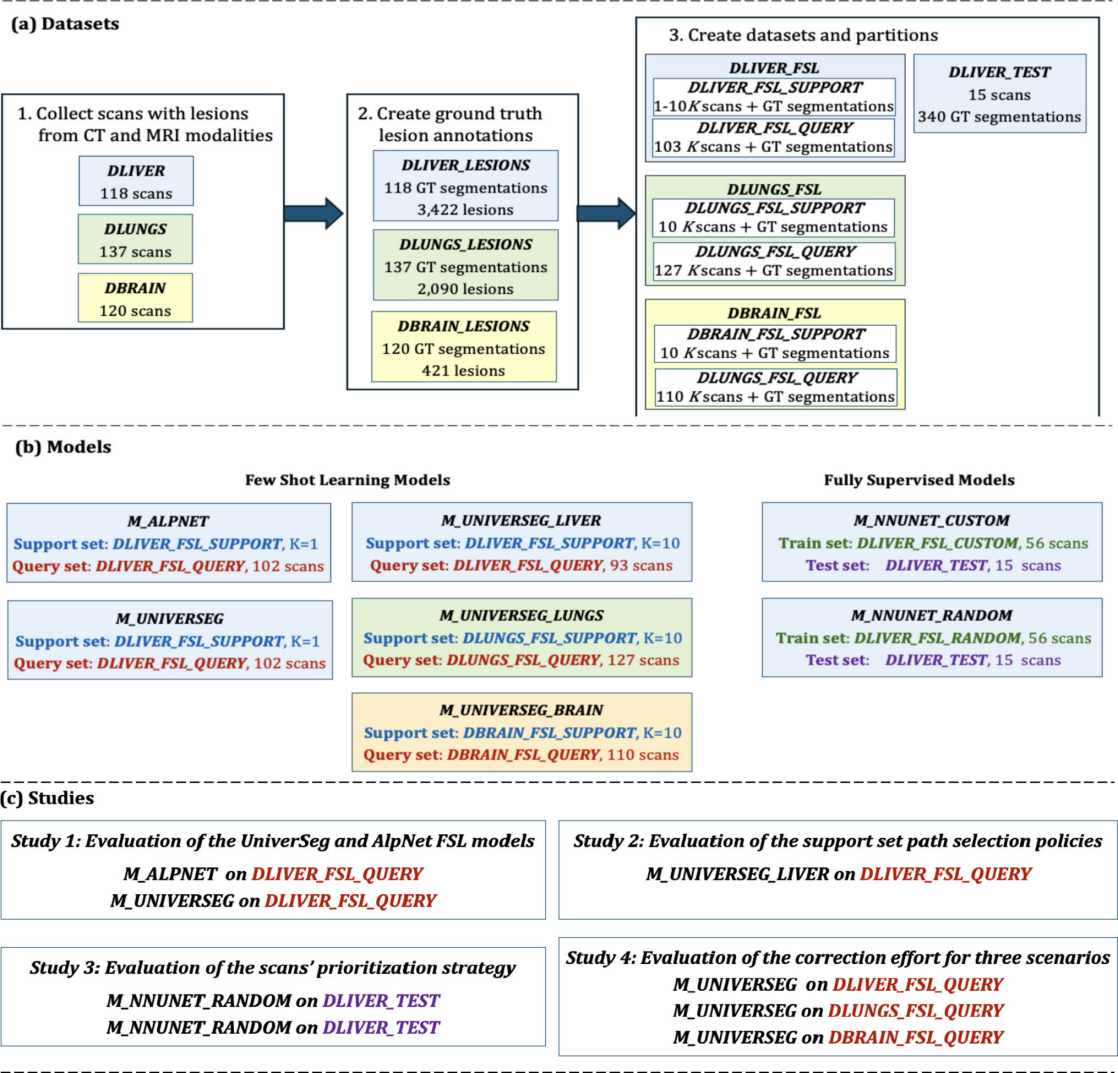
Flowchart illustrating: **a** the creation of the datasets, the ground truth annotations (GT), and the partition of the datasets into training, support, query, and test sets; **b** models and corresponding datasets used to train them, with their respective support/training and query/testing partition; **c**: experimental studies including the models and the datasets employed for their execution

Three datasets of liver, lung, and brain scans were created (Fig. 2): ***DLIVER*** (118 abdominal CECT scans), ***DLUNGS*** (137 chest CT scans), and ***DBRAIN*** (120 brain T1W-Gad MRI scans). Each dataset was partitioned into support, query and test sets. ***DLIVER*** was split into ***DLIVER_FSL*** (103 scans) for few-shot learning inference and ***DLIVER_TEST*** (15 scans) for final evaluation. ***DLIVER_FSL*** was further partitioned into ***DLIVER_FSL_SUPPORT*** with $K$=10 support scans and ***DLIVER_FSL_QUERY* (**103 scans) query scans. ***DLUNGS*** and ***DBRAIN*** are also partitioned into ***DLUNGS_FSL_SUPPORT*** and ***DBRAIN_FSL_SUPPORT*** with *K* = 10 support scans, and ***DLUNGS_FSL_QUERY*** (127 scans) and ***DBRAIN_FSL_QUERY*** (110 scans) query scans.

**Manual annotation:** Ground truth (***GT***) lesion annotations for the ***DLIVER*** and ***DLUNGS*** datasets were created by an expert radiologist; a senior neurosurgeon annotated the ***DBRAIN*** dataset. This resulted in the ***DLIVER-LESIONS***, ***DLUNGS-LESIONS***, and ***DBRAIN-LESIONS*** datasets, comprised of, 3,422 liver, 2,090 lung and 421 brain lesions, respectively. Of these, 252 liver, 283 lung, and 135 brain lesions were $\ge$ 10 mm. Each liver, lung, and brain scan included a mean (std) of 20.4 ± 7.9, 15.2 ± 19.1, and 3.5 ± 2.7 lesions. Lesions with < 30 voxels were excluded.

**Evaluation metrics:** Two types of metrics were used: (1) ***Lesion detection and segmentation***: ***Precision, Recall, F1-score*** for lesion detection. Computed lesions with at least one voxel overlap with the ground truth segmentation were considered true positives (***TP***); lesions with no overlap were considered false positives (***FP***). Lesions present in the ground truth but not computed were considered false negatives (***FN***). The ***Dice*** score was computed for each TP lesion and then averaged over all scans; the ***Dice_with_FN*** score was computed for all computed lesions, including FNs and FPs, to quantify the segmentation quality when the lesion detection precision and/or recall are low. (2) ***Estimated correction effort***: the effort required by a radiologist to correct the detected computed lesions and mark the missed ones. It is quantified with the number of mouse clicks required to correct a computed lesion: one mouse click to discard a FP and add a FN, and one mouse click for each FN erroneous pixel on the computed lesion contour.

We introduce three new metrics: ***false negative ratio*** (***FNR***) and ***false positive ratio*** (***FPR***)—the ratio between FN and FP lesions and all GT lesions, and the ***contour correction score*** (***CCS***), the ratio between the number of erroneously computed pixels and the GT pixels in the lesion contour. Lower ***CCS*** scores indicate lower correction effort of the lesion contour.

**Experimental studies:** We designed and conducted four experimental studies as follows. Study 1 evaluates the performance of the UniverSeg and ALPNet FSL models. Study 2 evaluates the clustering and random policies for the selection of support set patches. Study 3 evaluates the scans prioritization strategy for the UniverSeg model. Study 4 quantifies the estimated manual correction effort of the UniverSeg model results on the lesions in the liver, lungs and brain datasets. Studies 1–3 justify our choices of UniverSeg for FSL, of the support set patch selection clustering policy, and of the manual correction scan prioritization strategy on the liver lesions. Study 4 demonstrates the generality of our method on lesions in the liver, lungs, and brain scans on two imaging modalities.

## Results

*Study 1: Evaluation of the UniverSeg and ALPNet models.* The performance of the models was evaluated for 1-shot liver lesion detection and segmentation using fivefold cross-validation on the ***DLIVER_FSL*** dataset. We evaluated on a 1-shot setting since it best characterizes the performance of the FSL models. Both the ALPNet and UniverSeg models were built with the pre-trained weights published in the original papers [12, 13]. For each fold, one scan was randomly drawn from ***DLIVER_FSL***. The ground truth lesion segmentation was split into patches of size 128 × 128. The number of patches in the support set was set to 450 due to hardware limitations of the memory.

**Fig. 3:**
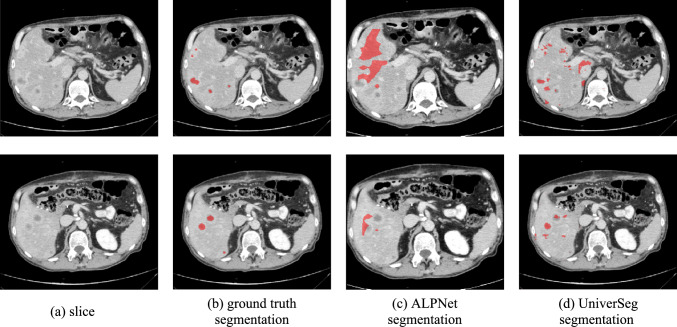
Illustration of Study 1. Two axial images from a CT scan (top and bottom) of the ground truth and the computed segmentations by the UniverSeg and ALPNet models of liver lesions (red). For this example, the precision and recall of the UniverSeg and ALPNet models were 1.00 and 0.18, 0.20 and 0.86 and 0.20, and the Dice scores were 0.13 and 0.57, respectively

The first 450 positive patches were included in the support set ***DLIVER_FSL_SUPPORT***. The patches of the remaining 102 scans in ***DLIVER_FSL*** were used for the query set ***DLIVER_FSL_QUERY***. Each support scan included a mean of 21 lesions. Table 1 lists the results. Figure 3 shows an example.

**Table 1:** Results of study 1

FSL Model		Detection			Segmentation	
		Precision	Recall	F1-score	Dice	Dice_with_FN
UniverSeg	Mean Std	0.61 (0.26)	0.59 (0.24)	0.43 (0.05)	0.49 (0.09)	0.30 (0.14)
ALPNet	Mean Std	1.00 (0.00)	0.31 (0.03)	0.45 (0.01)	0.05 (0.02)	0.03 (0.02)

Performance of the fivefold cross-validation of the UniverSeg and the ALPNet models in a one-shot setting ($K=1$) using 102 scans in the query set for each fold for lesions $\ge$ 10 mm in the ***DLIVER_FSL_QUERY*** dataset*.* Listed are the mean and standard deviations of the precision, recall, and F1-scores for lesion detection and the mean and standard deviation of the ***Dice*** and the ***Dice_with_FN*** scores. Bold numbers indicate the best-in-class performance

UniverSeg outperformed ALPNet for lesion detection, with a recall of 0.59 ± 0.24 vs. 0.31 ± 0.03 (90% higher); the perfect precision of ALPNet (1.00 ± 0.0) reflects the very low number of detected lesions. UniverSeg yields ***Dice*** and ***Dice_with_FN*** scores of 0.49 ± 0.09 vs. 0.05 ± 0.02 and 0.30 ± 0.14 vs. 0.03 ± 0.02, indicating that the ALPNet results are not useful. This justifies our choice of UniverSeg and illustrates the possible pitfalls of few-shot learning architectures.

**Fig. 4:**
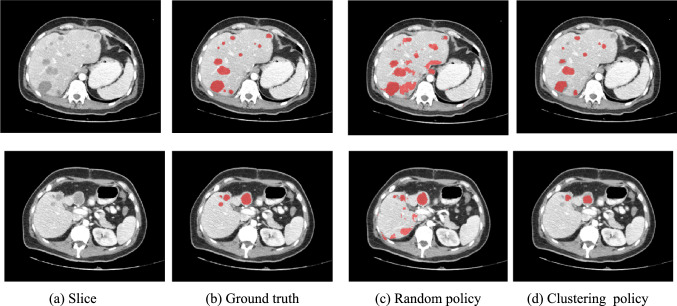
Illustration of Study 2. **a** Two axial images from a CT scan (top and bottom) of **b** the ground truth and **c**–**d** the computed lesion segmentations with UniverSeg with a support set of 10 scans, with **c** Random and **d** Clustering support set patch selection policies. For these examples, the precision and recall were 0.71 and 0.61, and the ***Dice*** score was 0.78 for both. For Study 3, the estimated correction effort ***FNR***, ***FPR*** and ***CCS*** were 0.39, 0.54 and 0.26 for all lesions, and 0.10, 0.26 and 0.18 for lesions $\ge$ 10 mm

*Study 2: Evaluation of the support set patch selection policies.* The performance of the UniverSeg model on liver lesions with the random and clustering support set patch selection policies was evaluated as follows. A set of ten support scans with a total of 200 lesions (~ 20 lesions/scan) in the ***DLIVER_FSL_SUPPORT*** liver dataset were split into 128 × 128 patches; 450 positive patches were selected with each policy. The model was tested on ***DLIVER_FSL_QUERY*** (93 scans) using fivefold cross-validation for lesions $\ge$ 10 mm. Table 2 lists the results. Figure 4 shows an example.

**Table 2:** Results of study 2

Support patches sampling method		Detection			Segmentation	
		*Precision*	*Recall*	*F1-score*	*Dice*	*Dice_with_FN*
*Random*	Mean (Std)	0.48 (0.37)	0.80 (0.25)	0.54 (0.35)	0.55 (0.28)	0.49 (0.29)
*Clustering*	Mean (Std)	0.58 (0.33)	0.82 (0.25)	0.58 (0.33)	0.55 (0.27)	0.50 (0.28)

Performance of the UniverSeg model with fivefold cross-validation on 93 query scans with a support set of patches sampled from 10 scans with the random and clustering policies for lesions $\ge$ 10 mm on the ***DLIVER_FSL_QUERY*** dataset*.* Listed are the mean (std) ***Precision***, ***Recall***, and ***F1-scores*** for lesion detection and ***Dice*** and the ***Dice_with_FN*** scores for lesion segmentation. Bold numbers indicate best-in-class performance

The clustering support set patch selection policy outperformed the random policy for lesions $\ge$ 10 mm, with a precision of 0.58 ± 0.33 vs. 0.48 ± 0.37 (20% higher), a recall of 0.82 ± 0.25 vs. 0.80 ± 0.25 (2.5% higher) and the same ***Dice*** and ***Dice_with_FN*** scores of 0.55 ± 0.27 and 0.50 ± 0.38. Note that the UniverSeg model performance with 10 support set examples is well below the that of fully supervised methods, with a reported precision and recall of 0.83 ± 0.24 (28% higher) and 0.95 ± 0.10 (13% higher) [14].

*Study 3: Evaluation of the scans prioritization strategy for manual correction*. The performance of the scans prioritization strategy for the correction of the lesion detection and segmentation results of UniverSeg was evaluated on the ***DLIVER*** dataset. The labels computed by the UniverSeg model were used to determine which subset of the scans should be manually corrected first with random and prioritization ordering. Two subsets of ***DLIVER_FSL_QUERY*** with 46 scans each were selected for each strategy. The ***DLIVER_FSL_SUPPORT*** dataset labels were used to generate initial segmentations from the ***DLIVER_FSL_QUERY*** dataset. Corrections were simulated by replacing erroneous computed labels with ground truth labels from the ***DLIVER*** dataset. The estimated correction effort was quantified with the ***FNR***, ***FPR*** and ***CCS*** metrics. Two nnU-Net models were trained with 56 scans – 10 scans from the support set and 46 scans with the corrected annotations. The nnU-Net models were evaluated on the ***DLIVER_TEST*** dataset.

Both nnU-Net models achieved similar performance in lesion detection and segmentation for lesions $\ge$ 10mm: perfect precision of 1.00, high recall of 0.80 ± 0.36 and 0.78 ± 0.35, F1-scores of 0.83 ± 0.35 and 0.81 ± 0.34 and Dice scores of 0.79 ± 0.24 and 0.79 ± 0.26 for the random and prioritization strategies, respectively. Table S1 (Supplemental Material) lists the results. Figure S1 (Supplemental Material) shows an example. These results, similar to those reported in [14], show that the nnU-Net models trained on sufficient, high-quality annotated datasets that were manually corrected have very similar performance than nnU-Net models trained on the same fully manually annotated scans.

The estimated correction effort for each strategy is listed in Table 3. The effort was significantly higher for the randomly selected scans than for those selected with the prioritization strategy. Prioritization reduced the detection correction effort for missed lesions (***FNR***) by 34% and for wrongly identified lesions (***FPR***) by 387%. For segmentation, 150 vs. 345 lesions (130% less) required revision and 68,677 vs. 367,270 (424% less) required lesion contour pixel correction. Moreover, while deleting a false positive lesion requires only a single mouse click on a CT slice, a high number of false positives might lead to disinterest and confusion of the radiologists, thereby causing them to abandon the task. There was no need for an additional iteration of the process, as the streamlined annotation process produced labeled examples requiring as few corrections as possible. Typically, one iteration suffices, with rare exceptions where the required performance is above the observer variability [15].

**Table 3:** Results of study 3

Strategy for the selection of manual correction scans	Estimated manual correction effort			
	Detection		Segmentation	
	*FP*	*FN*	*# of lesions*	*# of incorrect contour pixels*
*Random*	155	75	345	360,277
*Prioritized*	40	56	150	68,677

Estimation of the effort required by a radiologist for correcting the results of computed labels with the UniverSeg model with random and prioritized scan selection strategies. Listed are the number of mouse clicks required for a radiologist to correct the lesion detection and segmentation for both strategies. For lesion detection, a single mouse click is required for each FP and FN lesion. For lesion segmentation, listed are the number of lesions and the number of incorrect contour pixels that that require correction – one mouse click for each pixel

**Fig. 5:**
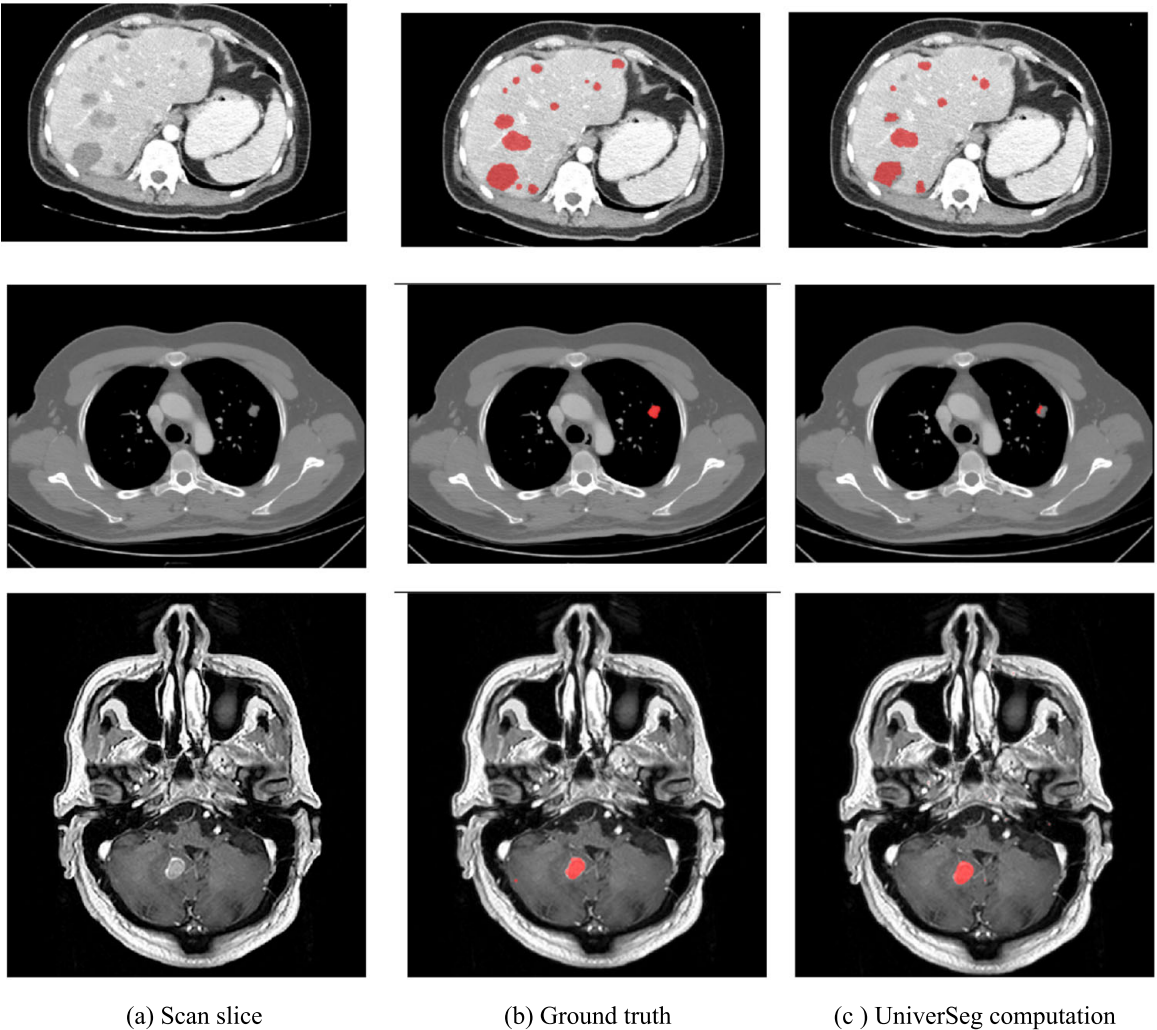
Illustration of the results of Study 4. Examples of the UniverSeg model results on liver (top), lung (middle), and brain (bottom) lesion in abdominal CECT, chest CT, and brain T1W-Gad MRI scans, respectively. The scores for the liver, lungs, and brain scans were as follows: the ***FNR*** was 0.39, 0.66 and 0.46, the ***FPR*** was 0.54, 0.87 and 0.46, the ***Dice*** score was 0.78, 0.50, and 0.75, and the ***CCS*** was 0.26, 0.50, and 0.28, respectively

*Study 4: Evaluation of the estimated effort required to correct annotations on three datasets*. This study quantifies the estimated effort required by a radiologist to manually correct the lesion detection and segmentation results computed with the UniverSeg model on the liver, lungs, and brain datasets. The effort is estimated by the mean number of mouse clicks required for removing false positive lesions, adding false negative lesions, and adjusting the lesion segmentation contours. A fivefold cross-validation of the number of clicks on ***DLIVER_FSL_QUERY*** (93 scans**)*, DLUNGS_FSL_QUERY*** (127 scans) and ***DBRAIN_FSL_QUERY*** (110 scans) datasets using a support set with patches sampled from 10 scans selected with the clustering policy was performed for all lesions and for lesions $\ge$ 10 mm. The detection includes the mean (std) ***FPR*** and ***FNR*** scores; the segmentation includes the mean (std) ***CCS***. Table 4 lists the results. Figure 5 shows an example.

**Table 4:** Results of study 4

Dataset	Lesion sizes		Detection		Segmentation
			*FPR*	*FNR*	*CCS*
*DLIVER*	All lesions	Mean (Std)	0.36 (0.31)	0.42 (0.23)	0.24 (0.20)
	$\ge$ 10 mm	Mean (Std)	0.27 (0.22)	0.18 (0.23)	0.25 (0.23)
*DLUNGS*	All lesions	Mean (Std)	0.93 (0.20)	0.95 (0.19)	0.64 (0.32)
	$\ge$ 10 mm	Mean (Std)	0.11 (0.27)	0.37 (0.46)	0.72 (0.45)
*DBRAIN*	All lesions	Mean (Std)	0.49 (0.01)	0.40 (0.37)	0.24 (0.17)
	$\ge$ 10 mm	Mean (Std)	0.30 (0.15)	0.08 (0.08)	0.25 (0.16)

Estimated correction effort scores of the liver, lung, and brain lesions (all and $\ge$ 10 mm) computed with the UniverSeg model with support set patch clustering selection. The results are the mean (std) for fivefold cross-validation estimation of the correction effort with the number of clicks required: ***FPR*** and ***FNR*** for lesion detection, ***CCS*** for lesion segmentation

For the liver lesions $\ge$ 10mm, the ***FNR*** was 0.18 ± 0.23, indicating that only 18% of the lesions need to be added to achieve a perfect recall vs. 82% more clicks required to detect them manually. The ***FPR*** was 0.27 ± 0.22, indicating that only 27% of the computed lesions had to be deleted. The ***CCS*** was 0.25 ± 0.23 for all lesions, indicating that 75% of the lesion contours in each slice were correct, thus reducing by × 4 the effort needed to segment a lesion manually. For lung lesions $\ge$ 10mm, the ***FNR*** was 0.40 ± 0.37, the ***FPR*** was 0.11 ± 0.27, and the ***CCS*** was 0.25 ± 0.23. For brain lesions $\ge$ 10 mm, the ***FNR*** was 0.08 ± 0.24, the ***FPR*** was 0.30 ± 0.15, and the ***CCS*** was 0.25 ± 0.16. These results indicate that while there is significant variability in the estimated effort for lesion detection and lesion contour correction, there is an overall significant reduction in the annotation effort. The results for all lesions show a higher correction effort and a similar distribution variability across organs.

***Limitations***: First, UniverSeg is designed and trained for 2D images; significant effort may be required for it to handle 3D images; however it can be replaced by a 3D FSL architecture when it becomes available. Second, UniverSeg inference requires 10–60 min/scan; however, the time required for generating labeled training datasets for the nnU-Net is performed offline. Third, the estimated correction effort does not take into account viewing and scan slice scrolling time, and the use of advanced editing tools; however, the metrics provide a quantitative measure with which various approaches can be objectively compared.

## Conclusion

The radiologists' annotation effort is a key bottleneck in the development and deployment of deep learning methods for volumetric medical image analysis. State-of-the-art supervised methods (nnU-Net) achieve superior results when trained with a sufficient number of high-quality annotated scans. Few-shot learning methods (UniverSeg) using a few annotated support set scans yield good results for large anatomical structures, e.g., organs and bones. However, they perform poorly for small structures, e.g., lesions, even for support sets with 10 scans (Study 2).

Our new method leverages the advantages of both few-shot learning models and supervised models while reducing the expected cost of manual annotation. It uses the UniverSeg model to produce an initial lesion voxel-level classification from a set of patches selected with a clustering strategy from a few annotated scans. This strategy yields labels that require fewer corrections than random selection or manual delineation (Study 2). A subset of the labeled scans is prioritized for review and correction by a radiologist. The resulting high-quality annotated scans, together with the support set scans, are then used to train an nnU-Net model, whose performance is evaluated with an annotated test set. Study 3 shows that the prioritization and random strategies yield similar nnU-Net performance; prioritization significantly reduces the estimated detection correction effort by 34% for missed lesions, by 387% for wrongly identified lesions, with 130% fewer lesion contour corrections and 424% fewer pixels to correct in the lesion contours. Study 4 demonstrates that our method is generic, applicable to various modalities (CT, CECT, and MRI), and to lesions in different organs, e.g., liver, lungs, and brain on a dataset of 375 scans with 5,933 lesions.

Our method effectively reduces the radiologists' annotation effort of small structures to produce sufficient high-quality annotated datasets to train deep learning models. It is generic and can be applied to a variety of lesions in various organs imaged with different modalities.

## Supplementary Information

Below is the link to the electronic supplementary material.

Supplementary file1 (PDF 839 KB)
