# Supplementary material for: Streamlining the annotation process by radiologists of volumetric medical images with few-shot learning
Source: Int J Comput Assist Radiol Surg. 2025 Jun 25;20(9):1863–73. doi: 10.1007/s11548-025-03457-3 (PMC12476431; doi:10.1007/s11548-025-03457-3)
Supplement: Supplementary file 1 — Supplementary file1 (PDF 839 KB) [file 11548_2025_3457_MOESM1_ESM.pdf]

Supplemental Material

Streamlining the Annotation Process by Radiologists in Volumetric Medical Images with Few-Shot Learning

Alina Ryabtsev[1] MSc, Richard Lederman[2] MD, Jacob Sosna[2] MD, Leo Joskowicz[1]* PhD

1. School of Computer Science and Engineering, The Hebrew University of Jerusalem, Israel.

2. Dept. of Radiology, Hadassah University Medical Center, Jerusalem, Israel.

4. Experimental results

Study 3: **Evaluation of the prioritization strategy for manual radiologist correction**.

nnU-Net models trained with support set patch selection		Detection			Segmentation	
		Precision	*Recall*	*F1-Score*	*Dice*	*Dice_with_FN*
Random	Mean<br>Std	**1.00**<br>(0.00)	**0.80**<br>(0.36)	**0.83**<br>(0.35)	**0.79**<br>(0.24)	**0.62**<br>(0.38)
Prioritized	Mean<br>Std	**1.00**<br>(0.02)	0.78<br>(0.35)	0.81<br>(0.34)	**0.79**<br>(0.26)	**0.61**<br>(0.39)

Table S1: Results of Study 3. Performance of two nnU-Net models, one trained on randomly and the other trained on prioritized manually corrected scans on the ***DLIVER_TEST*** dataset. The detection results include precision, recall, F1-score averages, and standard deviations. The segmentation results include the mean and std for the ***Dice*** and ***Dice_with_FN*** scores.

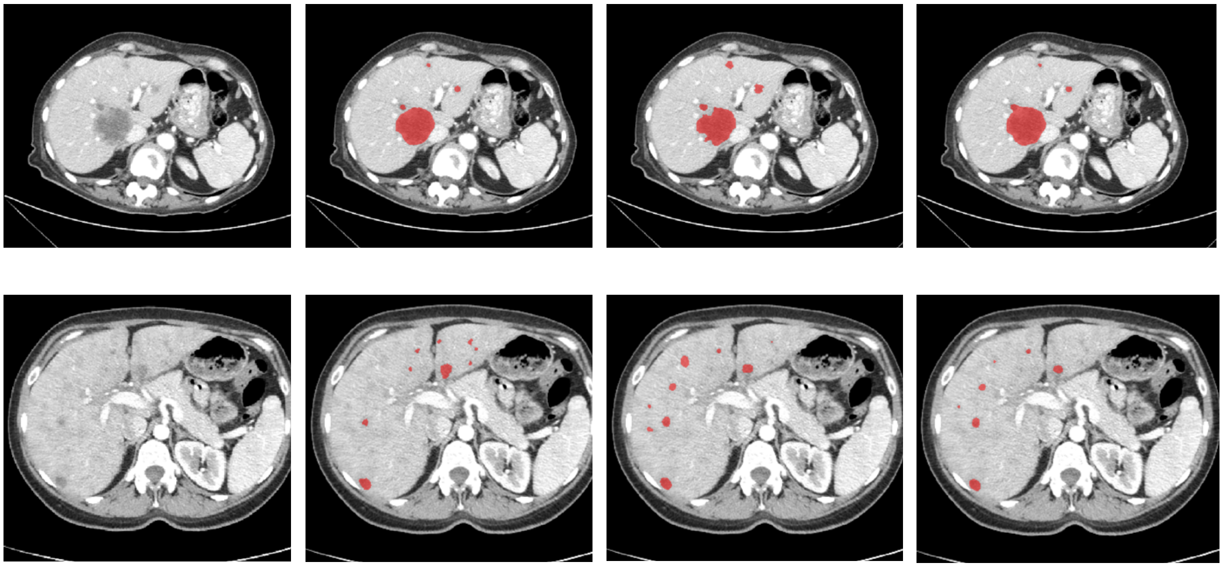

(a) slice | (b) Ground truth segmentation | (c) nnU-Net model trained on random selection | (d) nnU-Net model trained on prioritized selection

Fig. S1: Illustration of the results of Study 3. Two examples of test scan axial images with ground truth annotations and their corresponding liver lesions (red) computed by two nnU-Net models, trained on random and prioritized support set patch selection.
